# Notes and Comments

**Published:** 1895-01

**Authors:** 


### Notes and Comments.[1]

ERRATA.—On page 681 of the November number under the heading "Placing the Cap in Position," the word coronæ should have been printed *cornua*.

---

DR. J. AUSTIN DUNN, of 70 Dearborn Street, Chicago, has made a reduction in price of his valuable syringe from $2.00 to $1.50; with two platinum needles the charge will be $2.00. This syringe is one of the best of its kind in the market, and has been improved by the use of a glass barrel, enabling the operator to keep it antiseptically clean with less difficulty than with other material.

---

THE NECESSITY OF TRAINING.—A recent writer in the *Outlook* very truly says that it is on the higher planes and in the last stretches of achievement that training becomes almost indispensable. Supreme excellence is the condition of supreme success, and supreme excellence is the result of very high training of some sort. It is for this reason that so many men and women achieve a credit-

[1] The assistant editor solicits contributions for this department,—new methods, new remedies and formulas, or any short practical note which may prove of value to the practitioner or student. Address 212 South Fifteenth Street, Philadelphia.

able success, and so few reach the great heights and seize the great prizes.

There are hosts of good lawyers, but the great jurists are few; there are many excellent physicians and dentists, but the distinguished men are far from numerous; there are multitudes of useful and self-sacrificing ministers, but thinkers and leaders in the pulpit are rare; there are many good writers, but those who make literature in any generation are a very small group. The first and more immediate success may be won by character, industry, and good ability; the second and ultimate success is conditioned upon an excellence which involves an exacting and long-continued training.

---

A Plea for Enthusiasm.—At a meeting of the Missouri State Dental Association, Dr. A. H. Thompson presented a paper upon "The Duty of Young Men in the Dental Profession." There are few even now who lead, he said, and to whom the rank and file can look for inspiration and help. The Elijahs are being called away, and there are few Elishas to take up the mantles as they fall. We need men of genius, and to obtain them we must create environments and conditions favorable for their birth and development. This can only be done by general culture and a universal desire to advance beyond mediocrity. To this end the need of the times is enthusiasm, for, as has been well said, "the enthusiast must needs be honest, courageous, energetic,—and all these beget talent and ability." Therefore, cultivate enthusiasm, for that alone will lead to the awakening that will reveal to the soul its duty and its work.

---

Dentistry and Corsets.—One of the most successful dental surgeons in New York delicately suggests to his nervous patients, when there is nerve-rasping work to be done, that the easiest fitting garments that can be worn will increase the powers of endurance. Some of the baneful things he has to contend with in the operating-chair are new shoes, tight sleeves, high to choking collars, and, worst of all, corsets so tight-fitting that the patient, depending on high chest breathing, is in danger of suffocation when the rubber dam goes on to protect the excavated tooth preparatory to filling. —*N. Y. World.*

---

Antisepsis in Dentistry.—Dr. Shrady, editor of the *Medical Record*, every once in a while indulges in a "fling" at dentists and dentistry. His latest is an accusation as undeserved as usual, as at one sweep he accuses the profession of neglecting antisepsis. To one who reads dental literature and attends association meetings of late years it is surely known that the average dentist pays more attention to cleanliness of instruments and of persons than does the average physician. That surely is our own personal experience. If the egotistical editor of the *Medical Record* had taken pains to find the facts, we believe he would have arrived at the same conclusion.— *Western Dental Journal.*

